# Arcabouços para Uso em Bioengenharia de Vasos Sanguíneos: Quais as Perspectivas?

**DOI:** 10.36660/abc.20230341

**Published:** 2023-06-20

**Authors:** Leonardo Rufino Garcia, André Monti Garzesi, Flávio de Souza Brito, Marcello Laneza Felicio, Matheus Bertanha

**Affiliations:** 1 Hospital das Clínicas de Botucatu Faculdade de Medicina de Botucatu Universidade Estadual Paulista São Paulo SP Brasil Hospital das Clínicas de Botucatu e Faculdade de Medicina de Botucatu – Universidade Estadual Paulista (UNESP) – Serviço de Cirurgia Cardiovascular e Transplante Cardíaco, São Paulo, SP – Brasil; 2 Hospital das Clínicas de Botucatu Faculdade de Medicina de Botucatu Universidade Estadual Paulista São Paulo SP Brasil Hospital das Clínicas de Botucatu e Faculdade de Medicina de Botucatu – Universidade Estadual Paulista (UNESP) – Serviço de Cardiologia, São Paulo, SP – Brasil; 3 Hospital das Clínicas de Botucatu Faculdade de Medicina de Botucatu Universidade Estadual Paulista São Paulo SP Brasil Hospital das Clínicas de Botucatu e Faculdade de Medicina de Botucatu – Universidade Estadual Paulista (UNESP) – Serviço de Cirurgia Vascular, São Paulo, SP – Brasil

**Keywords:** Doenças Cardiovasculares, Mortalidade, Vasos Sanguíneos, Matriz Nuclear (Scaffold, Engenharia Tecidual, Bioengenharia/tendências, Celulas Tronco, Materiais Biocompatíveis

As doenças cardiovasculares constituem atualmente as principais causas de morbimortalidade no Brasil e no mundo.^1,2^ Seu tratamento é multimodal e tem como um dos alvos o combate à progressão do seu principal substrato fisiopatológico, a aterosclerose. As mudanças de estilo de vida, prescrição e uso de antiagregantes plaquetários, anticoagulantes e hipolipemiantes, tratamento de comorbidades como hipertensão arterial sistêmica e diabetes mellitus até revascularizações *lato sensu* de órgãos-alvo são importantes no controle de sua evolução clínica.

Entretanto, muitos pacientes ainda apresentam evolução desfavorável da aterosclerose apenas com manejo clínico e se tornam candidatos a procedimentos de revascularização. Dessa forma, é imprescindível a disponibilidade de substitutos vasculares para uso como enxertos, os quais podem ser autólogos ou sintéticos (artificiais). Por sua vez, técnicas endovasculares com o uso de *stents* podem ser utilizadas no tratamento de estenoses ou oclusões, de acordo com indicações clínicas específicas.

Nas cirurgias para revascularização do miocárdio os enxertos mais utilizados são os de artérias torácicas internas, veia safena magna e artéria radial.^3^ Alguns estudos já mostraram maior patência de enxertos arteriais sobre os venosos em longo prazo^4^ com reflexos clínicos importantes, notadamente a diminuição de eventos cardiovasculares após o procedimento, por exemplo. Contudo, em cerca de 30% dos casos há indisponibilidade de enxertos autólogos, tanto pela má qualidade ou pelo fato de já terem sido usados previamente,^5^ existência de veias ou artérias com calibres inadequados para uso como substituto vascular, ausência de enxertos vasculares artificiais coronarianos compatíveis e infecção em sítio cirúrgico ou a distância que possam contaminar enxertos sintéticos,^6^ comprometendo resultados cirúrgicos devido à revascularização incompleta^3^ ou complicações e falhas após a revascularização.

Em cirurgias vasculares que envolvam vasos de maior calibre, opções de enxertos artificiais têm uso mais amplo e difundido, destacando-se os de politetrafluoroetileno expandido (ePTFE) e os de polietileno tereftalato (PET ou Dacron). As principais vantagens proporcionadas são a força para suportar a pressão arterial sistêmica, inércia do ponto de vista químico, ampla variedade de extensões e calibres para adequação ao território vascular a ser tratado e a possibilidade de armazenamento para rápido uso.^5^ Existem, porém, desvantagens advindas do uso de enxertos vasculares artificiais. Lenta endotelização permite exposição da superfície hidrofóbica endoluminal com adsorção de proteínas plasmáticas ao enxerto e subsequente ativação e agregação plaquetárias com formação de trombos. Posteriormente desenvolve-se resposta imune, com infiltração macrofágica e expressão de citocinas inflamatórias que guiam a proliferação de células musculares lisas e o desenvolvimento de hiperplasia neointimal.^5^

Ainda existe, contudo, importante lacuna a ser preenchida quando há a necessidade de enxertos vasculares de diâmetros inferiores a seis milímetros nos casos de indisponibilidade de enxertos autólogos. Tendo esse desafio a solucionar, há duas estratégias sendo mais comumente investigadas pela bioengenharia de enxertos. Arcabouços ou *scaffolds* biológicos, que são compostos por matriz extracelular derivados de tecidos biológicos descelularizados, empregados nas condições tridimensionais naturais ou como matéria-prima para bioconstrução por engenharia de tecidos, em que células-tronco são depositadas e induzidas à diferenciação celular apropriada. Esses arcabouços podem ser obtidos de tecidos animais ou cadáveres e apresentam como vantagem possuir a composição básica das proteínas da matriz extracelular, bioatividade e arquitetura natural do tecido em três dimensões.^7,8^ Nesse sentido, Oliveira et al.^9^ apresentam a perspectiva de uso dos vasos de placenta bovina para produção de biomateriais descelularizados como uma fonte de biomateriais vasculares como perspectiva futura para engenharia de tecidos vasculares com resultados promissores.

Por sua vez, existem os arcabouços de origem sintética, como o ácido poliglicólico e o ácido poliláctico, disponíveis comercialmente, em que os enxertos são pré-fabricados a partir de polímeros naturais ou sintéticos,^5^ fabricados de forma tridimensional ou bioimpressos tridimensionalmente (células e os produtos de suporte são estruturados conjuntamente), os quais apresentam como vantagem não induzir reações imunogênicas.^10^

Entretanto, a engenharia de tecidos ainda apresenta alguns desafios a serem superados para que alguns conceitos possam ser aplicados na prática clínica: capacidade replicativa limitada das células implantadas, perda da atividade da telomerase de células somáticas adultas, propriedades mecânicas diferentes das dos vasos nativos, biocompatibilidade e cinética de degradação e remodelamento dos enxertos *in vivo,*^11^ capacidade limitada de promover o crescimento celular e de regular a extensão e força da adesão celular, atividade de crescimento, diferenciação celular e maturação para o fenótipo desejado, inibir a inflamação e à proliferação neointimal, inibir a trombogenicidade, sustentar a produção de matriz extracelular e permitir o carreamento de nutrientes de forma seletiva através da parede do vaso.^12^

A transposição da pesquisa experimental para a pesquisa aplicada em humanos requer o enfrentamento de muitas das dificuldades relatadas ainda em pesquisas *in vitro* e em pesquisas experimentais para, por fim, chegar ao tratamento de doenças.^13^ De qualquer forma, os inúmeros trabalhos científicos apresentados em todo o mundo demonstram um grande avanço no conhecimento da manipulação celular e bioengenharia de materiais, que podem ser projetados para serem multifuncionais e possivelmente programáveis, o que aponta para um futuro promissor, a médio prazo.^9^ O trabalho em questão corrobora essa afirmação.

## References

[1] Oliveira GM, Brant LC, Polanczyk CA, Malta DC, Biolo A, Nascimento BR (2022). Cardiovascular Statistics - Brazil 2021. Arq Bras Cardiol.

[2] Tsao CW, Aday AW, Almarzooq ZI, Alonso A, Beaton AZ, Bittencourt MS (2022). Heart Disease and Stroke Statistics-2022 Update: A Report From the American Heart Association. Circulation.

[3] Lawton JS, Holland JE, Bangalore S, Bates ER, Beckie TM, Bischoff JM (2022). 2021 ACC/AHA/SCAI Guideline for Coronary Artery Revascularization: Executive Summary: A Report of the American College of Cardiology/American Heart Association Joint Committee on Clinical Practice Guidelines. Circulation.

[4] Taggart DP, Benedetto U, Gerry S, Altman DG, Gray AM, Lees B (2019). Bilateral versus Single Internal-Thoracic-Artery Grafts at 10 Years. N Engl J Med.

[5] Moore MJ, Tan RP, Yang N, Rnjak-Kovacina J, Wise SG (2022). Bioengineering artificial blood vessels from natural materials. Trends Biotechnol.

[6] Bertanha M (2016). Perspectivas de uso de células-tronco em cirurgia vascular. J Vasc Bras.

[7] Bertanha M, Moroz A, Jaldin RG, Silva RA, Rinaldi JC, Golim MA (2014). Morphofunctional characterization of decellularized vena cava as tissue engineering scaffolds. Exp Cell Res.

[8] Bertanha M, Moroz A, Almeida R, Alves FC, Valério MJ, Moura R (2014). Tissue-engineered blood vessel substitute by reconstruction of endothelium using mesenchymal stem cells induced by platelet growth factors. J Vasc Surg.

[9] Oliveira TS, Smirnow I, Santee KM, Miglino MA, Barreto RSN (2023). Decellularized Vascular Scaffolds Derived from Bovine Placenta Blood Vessels. Arq Bras Cardiol.

[10] Niklason LE, Lawson JH (2020). Bioengineered human blood vessels. Science.

[11] Williams SK, Birla RK (2020). Tissue engineering solutions to replace contractile function during pediatric heart surgery. Tissue Cell.

[12] Song HH, Rumma RT, Ozaki CK, Edelman ER, Chen CS (2018). Vascular Tissue Engineering: Progress, Challenges, and Clinical Promise. Cell Stem Cell.

[13] Garcia LR, Polegato BF, Zornoff LA (2017). Challenges of Translational Science. Arq Bras Cardiol.

