# Supplementary material for: Arcabouços para Uso em Bioengenharia de Vasos Sanguíneos: Quais as Perspectivas?
Source: Arq Bras Cardiol. 2023 Jun 20;120(6):e20230341. [Article in Portuguese] doi: 10.36660/abc.20230341 (PMC10344349; doi:10.36660/abc.20230341)
Supplement: Translation

# Translation: Scaffolds for Use in Blood Vessel Bioengineering: What are the Prospects?

**Authors:** Leonardo Rufino Garcia, André Monti Garzesi, Flávio de Souza Brito, Marcello Laneza Felicio, Matheus Bertanha

**Affiliations:** 1 Brazil Hospital das Clínicas de Botucatu e Faculdade de Medicina de Botucatu – Universidade Estadual Paulista (UNESP) – Serviço de Cirurgia Cardiovascular e Transplante Cardíaco, São Paulo, SP – Brazil; 2 Brazil Hospital das Clínicas de Botucatu e Faculdade de Medicina de Botucatu – Universidade Estadual Paulista (UNESP) – Serviço de Cardiologia, São Paulo, SP – Brazil; 3 Brazil Hospital das Clínicas de Botucatu e Faculdade de Medicina de Botucatu – Universidade Estadual Paulista (UNESP) – Serviço de Cirurgia Vascular, São Paulo, SP – Brazil

**Keywords:** Cardiovascular Diseases, Mortality, Blood Vessels, Cell Nucleous (Scaffold, Tissue Engineering, Bioengineering/trends, Stem Cells, Biocompatible Materials

Cardiovascular diseases are currently the main causes of morbidity and mortality in Brazil and worldwide.^1,2^ Its treatment is multimodal and has as one of its targets the fight against the progression of its major pathophysiological substrate, atherosclerosis. Lifestyle changes, prescription and use of antiplatelet agents, anticoagulants, and hypolipidemic agents, treatment of comorbidities such as systemic arterial hypertension and diabetes mellitus, and even revascularization of target organs *lato sensu* are important in controlling its clinical evolution.

However, many patients still have an unfavorable evolution of atherosclerosis with only clinical management and become candidates for revascularization procedures. Thus, the availability of vascular substitutes for use as grafts is essential, which can be autologous or synthetic (artificial). In turn, endovascular techniques with stents can be used to treat stenoses or occlusions according to specific clinical indications.

In surgeries for myocardial revascularization, the most used grafts are those from internal thoracic arteries, great saphenous veins, and radial arteries.^3^ Some studies have already shown greater patency of arterial grafts over venous ones in the long term^4^ with important clinical outcomes, notably decreased cardiovascular events after the procedure. However, in approximately 30% of cases, autologous grafts are not available, either because of poor quality or because they have already been used previously,^5^ the existence of veins or arteries with inadequate calibers for use as a vascular substitute, absence of vascular grafts compatible with coronary targets and infection at the surgical site or at a distance that may contaminate synthetic grafts,^6^ compromising surgical results due to incomplete revascularization^3^ or complications and failures after revascularization.

In vascular surgeries involving larger caliber vessels, artificial graft options are more widespread and widely used, emphasizing expanded polytetrafluoroethylene (ePTFE) and polyethylene terephthalate (PET or Dacron). The main advantages are the strength to withstand systemic arterial pressure, inertia from a chemical point of view, a wide variety of extensions and calibers to adapt to the vascular territory to be treated, and the possibility of storage for quick use.^5^ However, there are disadvantages arising from using artificial vascular grafts. Slow endothelialization allows exposure of the endoluminal hydrophobic surface with adsorption of plasma proteins to the graft and subsequent platelet activation and aggregation with thrombus formation. Subsequently, an immune response develops, with macrophage infiltration and expression of inflammatory cytokines that guide the proliferation of smooth muscle cells and the development of neointimal hyperplasia.^5^

However, there is still a pivotal gap to be filled when there is a need for vascular grafts with diameters smaller than six millimeters in cases of unavailability of autologous grafts. Having this challenge to solve, two strategies are being most commonly investigated by graft bioengineering. Biological scaffolds, composed of extracellular matrix derived from decellularized biological tissues, are used in natural three-dimensional conditions or as raw material for bioconstruction by tissue engineering, in which stem cells are deposited and induced to appropriate cell differentiation. These scaffolds can be obtained from animal tissues or cadavers and have the advantage of having the basic composition of extracellular matrix proteins, bioactivity, and natural tissue architecture in three dimensions.^7,8^ In this sense, Oliveira et al.^9^ present the perspective of using bovine placental vessels to produce decellularized biomaterials as a source of vascular biomaterials as a future perspective for vascular tissue engineering with promising results.

In turn, there are scaffolds of synthetic origin, such as polyglycolic acid and polylactic acid, commercially available options in which the grafts are prefabricated from natural or synthetic polymers,^5^ manufactured three-dimensionally or three-dimensionally bio-printed (cells and support products are structured together), which have the advantage of not inducing immunogenic reactions.^9^

However, tissue engineering still presents some challenges to overcome so that some concepts can be applied in clinical practice: limited replicative capacity of implanted cells, loss of telomerase activity of adult somatic cells, mechanical properties different from those of native vessels, biocompatibility and kinetics of degradation and remodeling of grafts in vivo,^10^ limited ability to promote cell growth and to regulate the extent and strength of cell adhesion, growth activity, cell differentiation and maturation to the desired phenotype, inhibit inflammation and neointimal proliferation, inhibit thrombogenicity, sustain the production of extracellular matrix and allow the selective transport of nutrients through the vessel wall.^11^

The transposition of experimental to applied research in humans requires facing many of the difficulties reported *in vitro* research and experimental research to reach the treatment of diseases in clinical practice^12^ finally. In any case, the numerous scientific works presented worldwide demonstrate a great advance in the knowledge of cellular manipulation and bioengineering of materials, which can be designed to be multifunctional and possibly programmable, which points to a promising future in the medium term.^9^ The work in question corroborates this statement.
